# Adaptor Proteins Intersectin 1 and 2 Bind Similar Proline-Rich Ligands but Are Differentially Recognized by SH2 Domain-Containing Proteins

**DOI:** 10.1371/journal.pone.0070546

**Published:** 2013-07-25

**Authors:** Olga Novokhatska, Mykola Dergai, Liudmyla Tsyba, Inessa Skrypkina, Valeriy Filonenko, Jacques Moreau, Alla Rynditch

**Affiliations:** 1 Department of Functional Genomics, Institute of Molecular Biology and Genetics, Kyiv, Ukraine; 2 State Key Laboratory of Molecular and Cellular Biology, Institute of Molecular Biology and Genetics, Kyiv, Ukraine; 3 Department of Cell Signal Systems, Institute of Molecular Biology and Genetics, Kyiv, Ukraine; 4 Department of Molecular Mechanisms of Development, Jacques Monod Institute, Paris, France; Hungarian Academy of Sciences, Hungary

## Abstract

**Background:**

Scaffolding proteins of the intersectin (ITSN) family, ITSN1 and ITSN2, are crucial for the initiation stage of clathrin-mediated endocytosis. These proteins are closely related but have implications in distinct pathologies. To determine how these proteins could be separated in certain cell pathways we performed a comparative study of ITSNs.

**Methodology/Principal Findings:**

We have shown that endogenous ITSN1 and ITSN2 colocalize and form a complex in cells. A structural comparison of five SH3 domains, which mediated most ITSNs protein-protein interactions, demonstrated a similarity of their ligand-binding sites. We showed that the SH3 domains of ITSN2 bound well-established interactors of ITSN1 as well as newly identified ITSNs protein partners. A search for a novel interacting interface revealed multiple tyrosines that could be phosphorylated in ITSN2. Phosphorylation of ITSN2 isoforms but not ITSN1 short isoform was observed in various cell lines. EGF stimulation of HeLa cells enhanced tyrosine phosphorylation of ITSN2 isoforms and enabled their recognition by the SH2 domains of the Fyn, Fgr and Abl1 kinases, the regulatory subunit of PI3K, the adaptor proteins Grb2 and Crk, and phospholipase C gamma. The SH2 domains mentioned were unable to bind ITSN1 short isoform.

**Conclusions/Significance:**

Our results indicate that during evolution of vertebrates ITSN2 acquired a novel protein-interaction interface that allows its specific recognition by the SH2 domains of signaling proteins. We propose that these data could be important to understand the functional diversity of paralogous ITSN proteins.

## Introduction

Modern complexity of the eukaryotic membrane transport system is considered the result of paralogous gene expansion [1]. In mammals clathrin-mediated endocytosis (CME) is mediated by a machinery of proteins represented mostly by at least two paralogues. Among them are nucleators of the CME FCHo (Fer/Cip4 homology domain only) proteins, adaptor proteins Eps15 and intersectins (ITSNs), membrane-deforming amphiphysins, GTPases of the dynamin family, uncoating phosphatases synaptojanins, etc. All these paralogues are closely related within their protein families and to date the precise role of each family member remains unclear.

Members of the ITSN protein family, ITSN1 and ITSN2, serve as molecular scaffolds during CME [2]. These proteins are one of the key molecules in the earliest stages of internalization as they cluster the membrane-sculpting FCHo1/2 proteins that mark the assembly sites of clathrin-coated pits. ITSNs have the same domain organization and both are widely expressed in tissues [3], [4]. One more common feature of ITSNs is alternative splicing of their transcripts generating short and long isoforms. The short splice form (ITSN-S) comprises two EH (Eps15 homology) domains, a coiled-coil region (CCR) and five SH3 (Src 3 homology) domains. The long splice variant (ITSN-L) includes additionally the DH (Dbl homology) GDP/GTP exchange domain, the PH (plekstrin homology) and C2 domains. Most of these domains mediate protein-protein interactions necessary for the assembly of multiprotein complexes (reviewed in [5], [6]).

Recently, a link between endocytic abnormalities and neurodegenerative pathologies was proposed. Enlarged early endosomes were found in patients with Alzheimer’s disease, Down syndrome individuals and animal model of Down syndrome [7], [8]. ITSN1 was identified in searching for genes that could be responsible for these endocytic abnormalities [9], [10]. Its protein or mRNA levels are elevated in patients with Down syndrome and Alzheimer’s disease [9], [11], [12]. Moreover overexpression of ITSN1 increases aggregation of mutant huntingtin [13]. These observations led to extensive studies of ITSN1, which revealed its interactions with multiple proteins involved in endocytosis as well as in exocytosis, mitogenic signaling, actin cytoskeleton remodeling and cell survival (reviewed in [5], [6]). Another member of the ITSN protein family, ITSN2, has no established link with neurodegenerative pathologies. Instead, increased levels of ITSN2 transcripts are associated with prolonged disease-free survival of patients with breast cancer [14]. The data on ITSN2 are limited, and so far only a few of its protein partners were characterized [2], [15]–[17]. Most of the known ITSN2 functions are linked to the regulation of activity of the small GTPase Cdc42 in various cell types by its long splice variant that possesses a GDP/GTP-exchange domain [15], [18]–[20]. The ITSN1 long splice variant is neuron-enriched and participates in dendritic spine development and synaptic vesicle trafficking [21]–[23]. So far it remains obscure to what extent the roles of ITSNs are common in tissues where they are coexpressed. Thus, the aim of this work was to characterize ITSN2 and perform a comparative analysis with ITSN1. Here we show that ITSN2 is connected with ITSN1 as a component of common protein complexes and by common SH3 domain-interacting ligands. The distinctive feature of ITSN2 from ITSN1 is its EGF-dependent interaction with SH2 domains of certain signaling molecules that is regulated presumably by tyrosine phosphorylation.

## Materials and Methods

### Ethic Statement

This study was carried out in strict accordance with the Guidelines for Care and Use of Animals in Research of the National Academy of Sciences of Ukraine. The protocol was approved by the Bioethical Committee of the Institute of Molecular Biology and Genetics.

### Expression Constructs

The constructs encoding GST-fused SH3 domains of ITSN1 were described previously [24], [25]. The cDNAs of human ITSN2-S and sprouty 2 (SPRY2) were kind gifts of Dr. S. de la Luna (Barcelona, Spain) [26], [27]. The full coding sequence of ITSN2-S was subcloned into the pEGFP-C1 vector (Clontech) and the pcDNA4His/Max vector (Invitrogen) with an Omni tag sequence. The coding regions of the SH3 domains of ITSN2, namely the SH3A (residues 749–826), SH3B (residues 891–964), SH3C (residues 976–1047), SH3D (residues 1044–1127), SH3E (residues 1116–1193) and SH3(A–E) domains (residues 749–1193), were subcloned into the pGEX-4T-3 vector (GE Healthcare) that encodes a GST tag. The coding sequence corresponding to amino acids 349–499 of ITSN2 was subcloned into the pET28b vector (Novagen). The coding region of the cytoplasmic domain of Sema6A (residues 650–1021) was subcloned into the pcDNA4His/Max vector (Invitrogen) with an Omni tag. Sequences encoding the SH2 domains of Grb2 (residues 57–157), Crk (residues 5–127), Itk (residues 246–363), Fgr (residues 136–251), Fyn (residues 143–255), Abl1 (residues 140–244), PI3KR1-N (residues 327–437), PI3KR1-C (residues 617–724) and PLCg1 (residues 542–766) were subcloned into the pGEX-4T-3 vector (GE Healthcare).

Expression constructs encoding the full-length Reps1, the proline-rich domains of synaptojanin 1 (residues 1003–1558) and N-WASP (residues 22–459), the central domain of CdGAP (residues 174–683), mCherry-ITSN1-S and GFP-tagged ITSN1-S were described previously [24], [28]. cDNA of Flag-tagged Numb was kindly provided by Prof. P. P. Di Fiore (Milan, Italy) [29]. Myc-tagged POB1 was a kind gift of Dr. E. Santonico (Roma, Italy) [30].

### Antibodies

Rabbit polyclonal antibodies against the EH2 domain of human ITSN1 (anti-ITSN1) were described previously [25]. Mouse polyclonal antibodies anti-ITSN1/m were raised against the same immunogen comprising residues 214–291 of ITSN1. Polyclonal antibodies against the CCR of ITSN2 were produced in rabbits immunized with the recombinant His-tagged protein comprising amino acid residues 349–499 of human ITSN2.

Polyclonal SOS1 (C-23): sc-256 and dynamin 1 (C-16): sc-6402 antibodies, and monoclonal anti-Omni (D-8): sc-7270, anti-Myc (9E10): sc-40, anti-phosphotyrosine (PY 99): sc-7020 antibodies were purchased from Santa Cruz Biotechnology. Monoclonal anti-Flag clone M2 and anti-Intersectin/ESE-1 were purchased from Covance, Sigma and BD Biosciences, respectively.

### Protein Expression, Binding Assays and Western Blot Analysis

The recombinant His- or GST-fused proteins were expessed in *Escherichia coli* and affinity purified using Ni-NTA Agarose (Qiagen) or Sepharose 4B (GE Healthcare) according to the manufacturer’s instructions. GST-tagged proteins (5–20 µg) coupled to beads or GST alone were incubated for 2 h at 4°C with lysates of human cell lines or mouse tissues. Cell and tissue lysates were prepared in extraction buffer containing 20 mM Tris-HCl pH 7.5, 0.5% NP40, 150 mM NaCl, 10% glycerol, 1 mM Na_3_VO_4_ and protease inhibitor cocktail (Roche). The beads were then washed with extraction buffer three times at 4°C and boiled in Laemmli buffer (150 mM Tris-HCl pH 6.8, 2.5% glycerol, 10% SDS, 3% β-mercaptoethanol and 0.5% bromophenol blue). Proteins were resolved in SDS-PAGE, transferred to nitrocellulose membranes (Bio-Rad) and probed with appropriate antibodies for 1 h at room temperature. Detection was performed using ECL reagents. Chemiluminescence was captured with Molecular Imager ChemiDoc™ XRS+ (BioRad). Signal intensities were quantified using the ImageLab™ software. The data were processed with OriginPro software.

### Immunoprecipitation

For immunoprecipitation (IP), the cells were lysed in IP buffer (20 mM Tris-HCl pH 7.5, 0.5% NP40, 150 mM NaCl, 10% glycerol, 1 mM Na_3_VO_4_ and protease inhibitor cocktail). The cell lysate was mixed with antibodies and protein A/G PLUS-Agarose (Santa Cruz Biotechnology) prewashed in IP buffer. After incubation for 2 h at 4°C the beads were washed three times with IP buffer. Bound proteins were eluted by boiling in Laemmli sample buffer and analysed by SDS-PAGE and Western blotting.

### Cell Culture and Transfection

The HEK293, HeLa, MCF-7 and MDA-MB-231 cell lines were obtained from Bank of cell lines of the R. E. Kavetsky Institute of Experimental Pathology, Oncology and Radiobiology, NASU (Ukraine). The cells were maintained in DMEM (Dulbecco’s modified Eagle’s medium) supplemented with 10% fetal calf serum, 50 U/ml penicillin and 100 µg/ml streptomycin. The cells were transiently transfected with the JetPEI (polyethyleneimine, Polyplus Transfection) and processed 24 h after transfection.

### Immunofluorescence and Confocal Microscopy

Immunostaining of the cells was performed as described previously [31]. For immunofluorescence, Texas Red-conjugated goat anti-rabbit (Vector Laboratories Inc.), Alexa 488-conjugated goat anti-rabbit or Alexa 488-conjugated donkey anti-mouse secondary antibodies (Molecular Probes) were used. Confocal images were taken using a Zeiss LSM510 microscope. ImageJ software was used to calculate the Pearson’s coefficient of correlation.

### Sequence Analysis and 3D Modeling

Sequences were obtained from GenBank and analyzed using Vector NTI 8.0 (Invitrogen). Prediction of protein-protein interactions was carried out with Scansite [32]. Homology-based models of the SH3 domains of ITSN1 were built with MODELLER 9 v7 [33] using the resolved structures of the ITSN2 SH3 domains (Protein Data Bank IDs: 1uff.pdb, 1j3t.pdb, 1uhf.pdb, 1 ue9.pdb, 1 udl.pdb). Structures were assessed with MolProbity service [34]. The models obtained were refined using molecular dynamics tools of the MODELLER software.

### Statistical Analysis

All data are presented as mean ±SEM. Statistical analyses were performed using OriginPro 7.5 (OriginLab Corporation, USA). Mean values were compared using a Student’s unpaired t test. Statistical significance was accepted when P<0.05.

## Results

### ITSN1 and ITSN2 form a Complex in Cells

**Figure 1:**
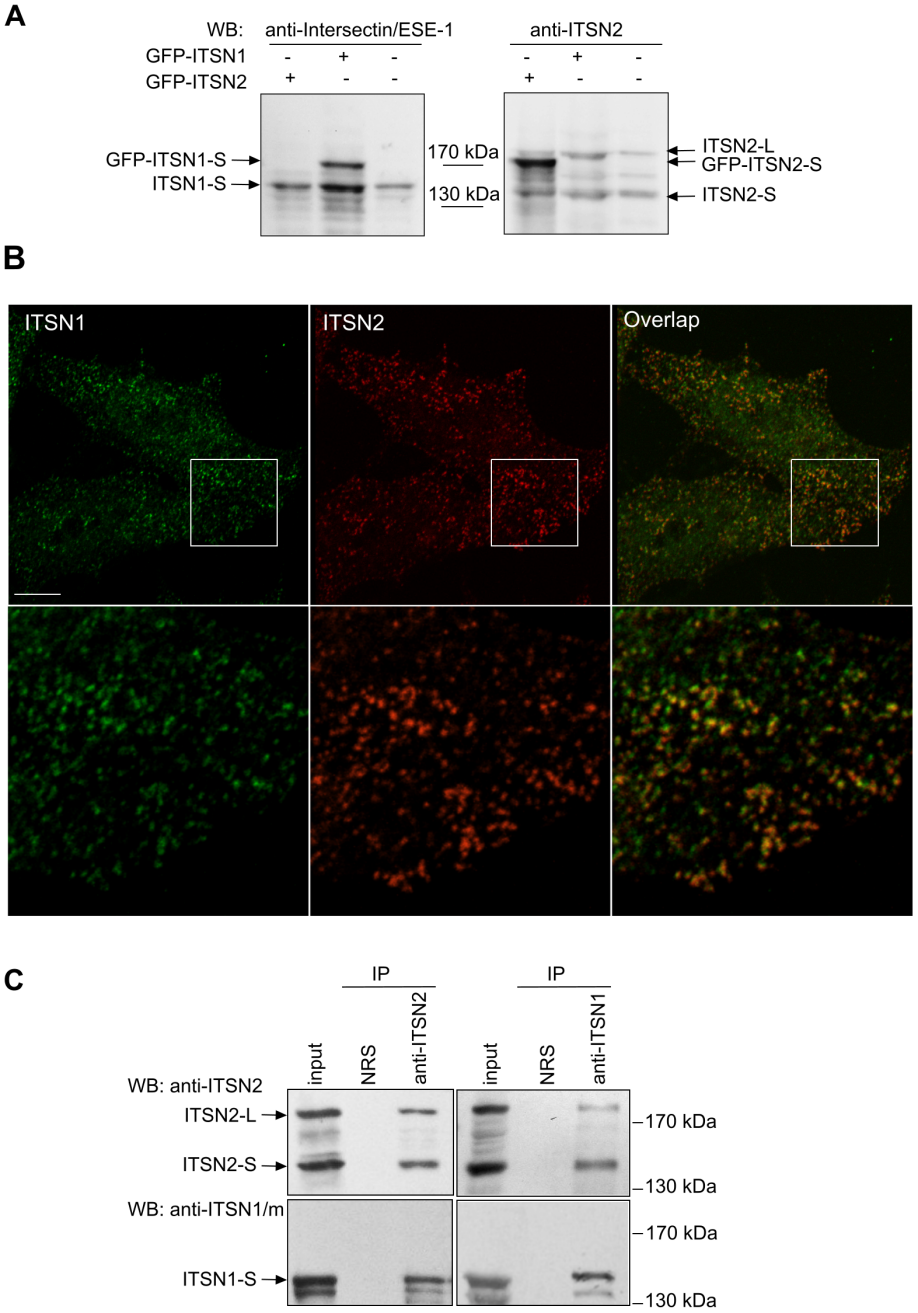
ITSN1 and ITSN2 are associated in cells. (A) Characterization of the anti-ITSN2 antibodies produced. Nontransfected HEK293 cells or cells expressing GFP-ITSN1-S or GFP-ITSN2-S were lysed 24 h post-transfection. Total cell lysates were resolved by SDS-PAGE with subsequent immunoblot analysis using the anti-ITSN2 antibodies obtained or the commercially available anti-Intersectin/ESE-1. (B) HEK293 cells were plated on coverslips and fixed. Endogenous ITSNs were stained with anti-ITSN1/m and anti-ITSN2 antibodies, and visualized with Alexa 488-conjugated or Texas Red-conjugated secondary antibodies, respectively. Higher magnification of the area enclosed by a rectangle is shown below each image. Scale bar: 10 µm. (C) Lysates of HEK293 cells were subjected to immunoprecipitation using anti-ITSN2 antibodies (left panels). Conversely immunoprecipitation was performed using rabbit polyclonal antibodies against ITSN1 (right panels). In both cases immunoprecipitated material was probed with antibodies specific to ITSN1 and ITSN2. Representative data from three independent experiments are shown. IP, immunoprecipitation; NRS, normal rabbit serum; WB, Western blotting.

To study whether ITSN2 can function in cellular compartments distinct from those of ITSN1, we compared the intracellular distribution of these proteins using newly generated antibodies against ITSN2. The immunogenic region was predicted within the CCR of ITSN2 that has the lowest level of homology with ITSN1. The polyclonal antibodies obtained specifically detected the short and long isoforms of ITSN2 in HEK293 cell lysates and demonstrated no cross-reactivity with ITSN1 (Figure 1A). Intracellular distribution of the ITSN1 protein was determined using anti-ITSN1/m antibodies. These antibodies recognized ITSN1 and did not detect ITSN2 (Figure S1A).

Immunofluorescence analysis using anti-ITSN1/m and anti-ITSN2 antibodies demonstrated that endogenous proteins colocalized in HEK293 cells (Pearson’s correlation coefficient, 0.67±0.06, n = 4; Figure 1B). Similar results were obtained with overexpressed Omni-ITSN2-S and mCherry-ITSN1-S proteins (Pearson’s correlation coefficient, 0.79±0.6, n = 4; Figure S2A). To investigate whether ITSN1 and ITSN2 could be involved in the same protein complexes, coimmunoprecipitation was performed. ITSN1-S was detected in immunoprecipitates obtained with anti-ITSN2 antibodies from lysate of HEK293 cells (Figure 1C, left panels). Both isoforms of ITSN2 coprecipitated with ITSN1-S using anti-ITSN1 antibodies (Figure 1C, right panels) arguing for the association of these adaptors. These data were confirmed by immunoprecipitation of endogenous ITSN1-S using anti-Omni antibodies from lysates of cells expressing Omni-ITSN2-S (Figure S2B). The specificity of anti-ITSN1 and anti-ITSN2 antibodies binding was confirmed using a competition assay (Figure S1B, C).

Thus, members of the ITSN protein family are predominantly colocalized and form a complex in cells.

### ITSNs Interact with Common Protein Partners via the SH3 Domains

To date, twenty-nine protein partners were reported for ITSN1 [5], [6], [35]–[37] and only five were shown for ITSN2 using biochemical methods [2], [15]–[17]. The major platform for protein-protein interactions of ITSN molecules is the tandem of five SH3 domains that binds twenty-two of twenty-nine ITSNs partners. Regarding the fact that ITSNs have the same domain organization, a similar subcellular distribution and are components of common complexes, we investigated to what extent pools of their binding partners could be different.

**Figure 2:**
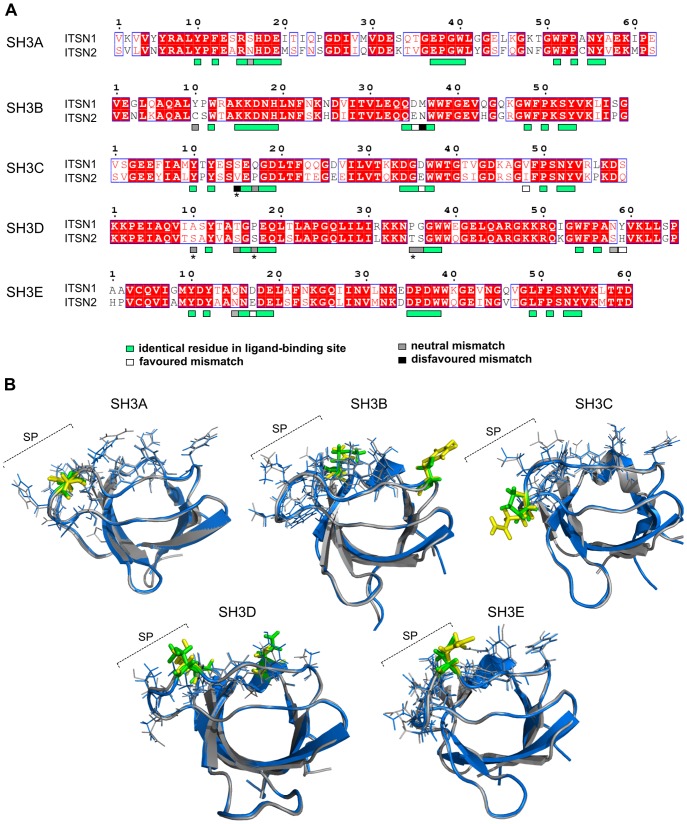
Comparison of ligand-binding sites of the SH3 domains of ITSN1 and ITSN2. (A) Alignment of protein sequences of the human ITSNs SH3 domains. The alignment was generated using the ClustalW algorithm. Identical residues are highlighted in red, and homologous amino acids are shown in red letters. Amino acid residues that form ligand-binding sites of the SH3 domains are indicated by boxes, mismatches within these regions are shown by boxes of different colours. Amino acid residues marked with an asterisk are not conserved in ITSN1 and ITSN2 orthologues. (B) Merged structures are shown for each SH3 domain pair in blue and grey for ITSN1 and ITSN2, respectively. Amino acid residues that form ligand-binding sites of the SH3 domains and are identical in ITSNs or not conserved in orthologues are depicted as lines. Divergent residues involved in ligand binding of the ITSN1 and ITSN2 SH3 domains are depicted in yellow and green, respectively. Amino acid mismatches regarded as favoured are not shown in colour. SP, specificity pocket of the SH3 domain ligand-binding site.

To answer this question the structures of the SH3 domains of ITSN1 and ITSN2 were compared searching for differences in their ligand-binding sites. Previous analyses of SH3 domains bound to their ligands [38] permitted us to assign residues implicated in ligand-binding for the ITSNs SH3 domains (Figure 2A). The SH3 domains of human ITSNs share high levels of identity from 61% to 77% (Table 1). Unexpectedly, the frequency of mismatches within ligand-binding sites compared to that of the rest of the domain was different. This parameter was seven times lower for the SH3A domains, two times lower for the SH3B, SH3C and SH3E domains, and almost two times higher for the SH3D domains. Thus, the SH3A domains, despite having gained mismatches during evolution, kept their binding sites unaffected whereas ligand-binding sites of the SH3D domains acquired mismatches more rapidly than the rest of the domain. It should be mentioned that the SH3A domains are the most divergent whereas the SH3D domains have the highest level of identity among ITSNs SH3 domains. Differences in ligand-binding sites of the SH3 domains are summarized in Table 1.

**Table 1:** Differences within ligand-binding sites of the SH3 domains of human ITSN1 and ITSN2.

Domain	Identity	Number of mismatches		Mismatch¥	Position	Substitution preference¶
		within LBS §	beyond LBS§			
SH3A	61.0%	1	26	S → N	16	Neutral
SH3B	74.6%	3	16	Y → C	10	Neutral
				D → E	35	Favoured
				M → N	36	Disavoured
SH3C	66.1%	4	22	S → V*	15	Disavoured
				P → Q	17	Neutral
				D → E	36	Favoured
				V → I	48	Favoured
SH3D	76.9%	7	15	A → S*	10	Neutral
				T → S	15	Neutral
				P → S*	17	Neutral
				P → T*	35	Neutral
				G → S	36	Neutral
				N → S	58	Neutral
				Y → H	59	Favoured
SH3E	75.9%	2	16	Q → N	15	Neutral
				D → E	17	Favoured

§ LBS (ligand-binding site) of the SH3 domain consists of 15 amino acid residues [38];

¥ ITSN1 → ITSN2;

¶ for intracellular proteins according to Betts and Russell [42];

* mismatch is not conserved in orthologues.

To get insight in the effect of each mismatch on binding, we compared the 3D structures of the human SH3 domains. For this, resolved structures of the ITSN2 SH3 domains were used, as well as the corresponding models obtained for ITSN1 in the present study and previously [39]. The ligand-binding site of the SH3 domain consists of two hydrophobic pockets formed by conserved aromatic residues and a third negatively charged specificity pocket composed of residues from the n-Src and RT loops [40], [41]. For all SH3 domains, ligand-binding site mismatches were predominantly located in the third specificity pocket (Figure 2B) and conserved in organisms ranging from fish to humans (Figure S3). Mismatches were classified as favored, neutral or disfavoured according to the substitution preferences for intracellular proteins [42].

The SH3A, SH3C and SH3E domain pairs had the most similar ligand-binding sites with one neutral mismatch each. Additionally, the SH3C and SH3E pairs possessed two and one favoured mismatch, respectively. Mismatch in the SH3C pair S(15)V was not taken into consideration as it was not conserved. In reptiles and birds the SH3C domains of ITSN2 contained serine in this position similarly to ITSN1 (Figure S3). Since most protein partners interact with ITSN1 via the SH3A, SH3C and SH3E domains (for a review, see [5]), we suggest that these domains in ITSN2 will predominantly bind the same ligands.

Within the SH3B pair three mismatches that belonged to favoured, neutral and disfavoured groups were found. Here the neutral mismatch Y(10)C was located in the hydrophobic pocket and could affect the strength of the contact with the proline residue in the ligand. Cysteine is a hydrophobic residue but is unable to properly form stacking interactions with proline. The disfavoured M(36)N mismatch could play a role in defining target specificity of the SH3B domain.

The SH3D pair gained seven mismatches, six of which were neutral and one considered as favoured. These mismatches were found in positions responsible both for binding and for target specificity. Three neutral mismatches A(10)S, P(17)S and P(35)T were not conserved (Figure S3) and were not taken into consideration. However, it should be noted that the SH3D domain of ITSN1 prefers to bind to noncanonical based-rich motifs rather than to PXXP [43] demonstrating the prevalence of electrostatic over hydrophobic interactions in its ligand-binding. Therefore, it is hard to accurately predict the effect of the differences observed within ligand-binding sites of the SH3D domains on binding to protein partners.

**Figure 3:**
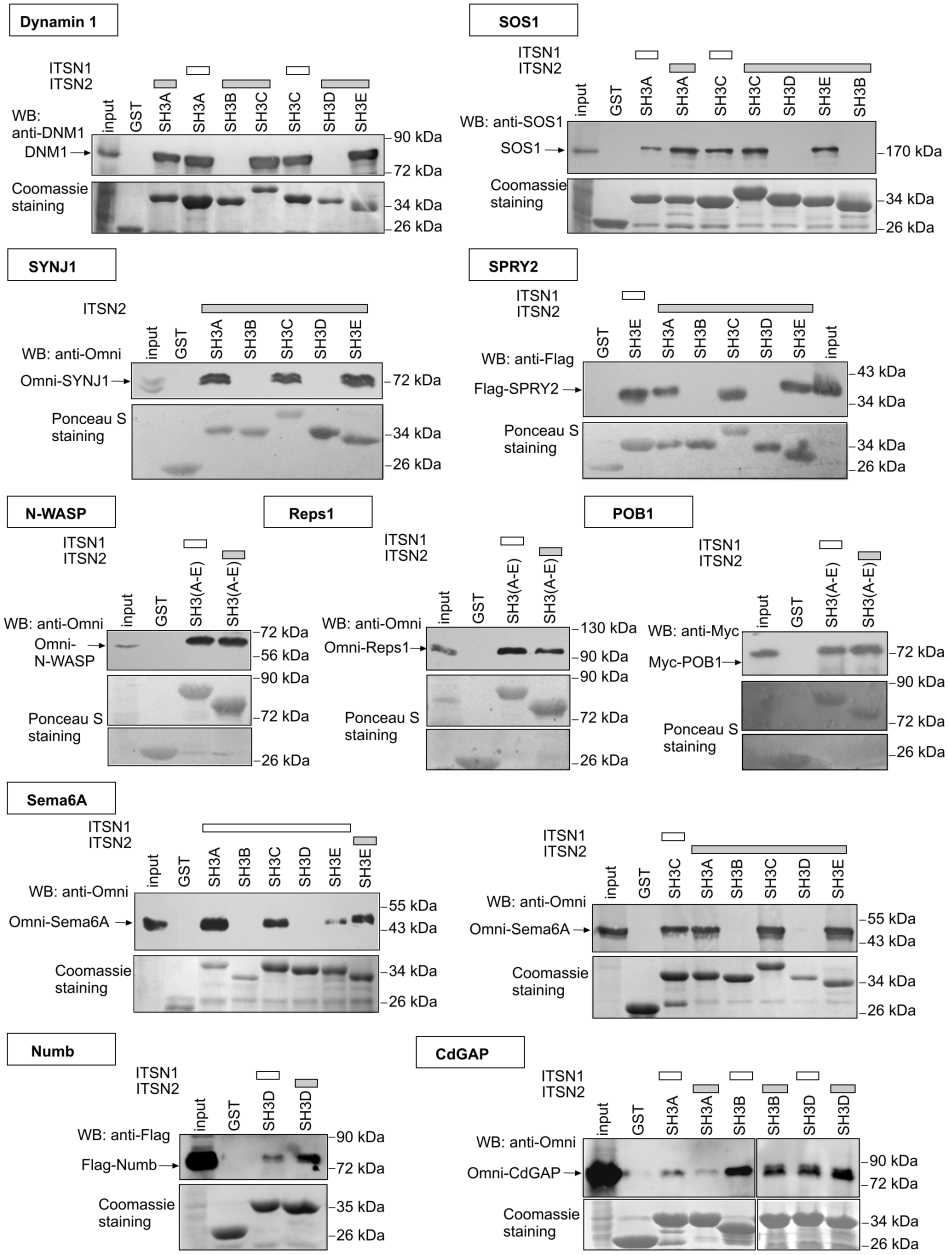
ITSNs have common SH3 domain interactors. The GST-fused SH3 domains of ITSN1 and ITSN2 were bacterially expressed and affinity purified. The GST-SH3 domains or GST alone (control) immobilized on glutathione beads were incubated with lysate of mouse brain (for dynamin 1 and SOS1) or HEK293 expressing Omni-synaptojanin 1 (SYNJ1), Omni-Reps1, Omni-N-WASP, Omni-CdGAP, Omni-Sema6A, Flag-SPRY2, Flag-Numb or Myc-POB1. Bound proteins were separated by SDS-PAGE and detected by immunoblotting with antibodies against dynamin 1 (DNM1), SOS1 or tags. GST-SH3 domains of ITSN1 were used as positive control of binding. GST-fused proteins were visualized by Coomassie or Ponceau S staining. The experiments were performed at least twice, and representative data are shown. WB, Western blotting.

To test the prediction experimentally, the interaction of ITSN2 with ten proteins was investigated, eight of which are known to bind ITSN1 and two that are identified as novel ITSNs interactors (Figure 3). Among the well-established protein partners of ITSN1 were GTPase dynamin 1 that drives fission of endocytic vesicles, phosphoinositide phosphatase synaptojanin 1 involved in vesicle uncoating, the exchange factor for Ras GTPase SOS1 protein, the regulator of receptor tyrosine kinase signaling SPRY2, the endocytic adaptor Reps1, the regulator of actin polymerization N-WASP, Numb implicated in dendritic spine development, and the Cdc42-inactivating protein CdGAP. The endocytic adaptor POB1 and Sema6A implicated in axon guidance were predicted as novel SH3 domain ligands using the Scansite service.

Using GST pull-down assay we showed interaction of dynamin 1, synaptojainin 1, SOS1, SPRY2 and a novel partner Sema6A with the SH3A, SH3C and SH3E domains of ITSN2 similarly to ITSN1. The SH3A, SH3B and SH3D domains of ITSN2, were able to pull down CdGAP as do those of ITSN1. Furthermore, interaction of the ITSN2 SH3D domain with Numb, a specific ligand of the ITSN1 SH3D domain, was demonstrated. Three more proteins, N-WASP, Reps1 and a novel partner POB1, also bound the SH3(A-E) domains of ITSN1 and ITSN2. Thus, no differential binding between the respective SH3 domains of ITSN1 and ITSN2 was observed.

Data from the comparison of ligand-binding sites and *in vitro* binding experiments suggest that the SH3 domains of ITSNs bind predominantly similar ligands. Certainly, the possibility of specific partners for the certain SH3 domains can not be excluded.

### ITSN2 Undergoes phosphorylation of Tyrosine Residues

**Figure 4:**
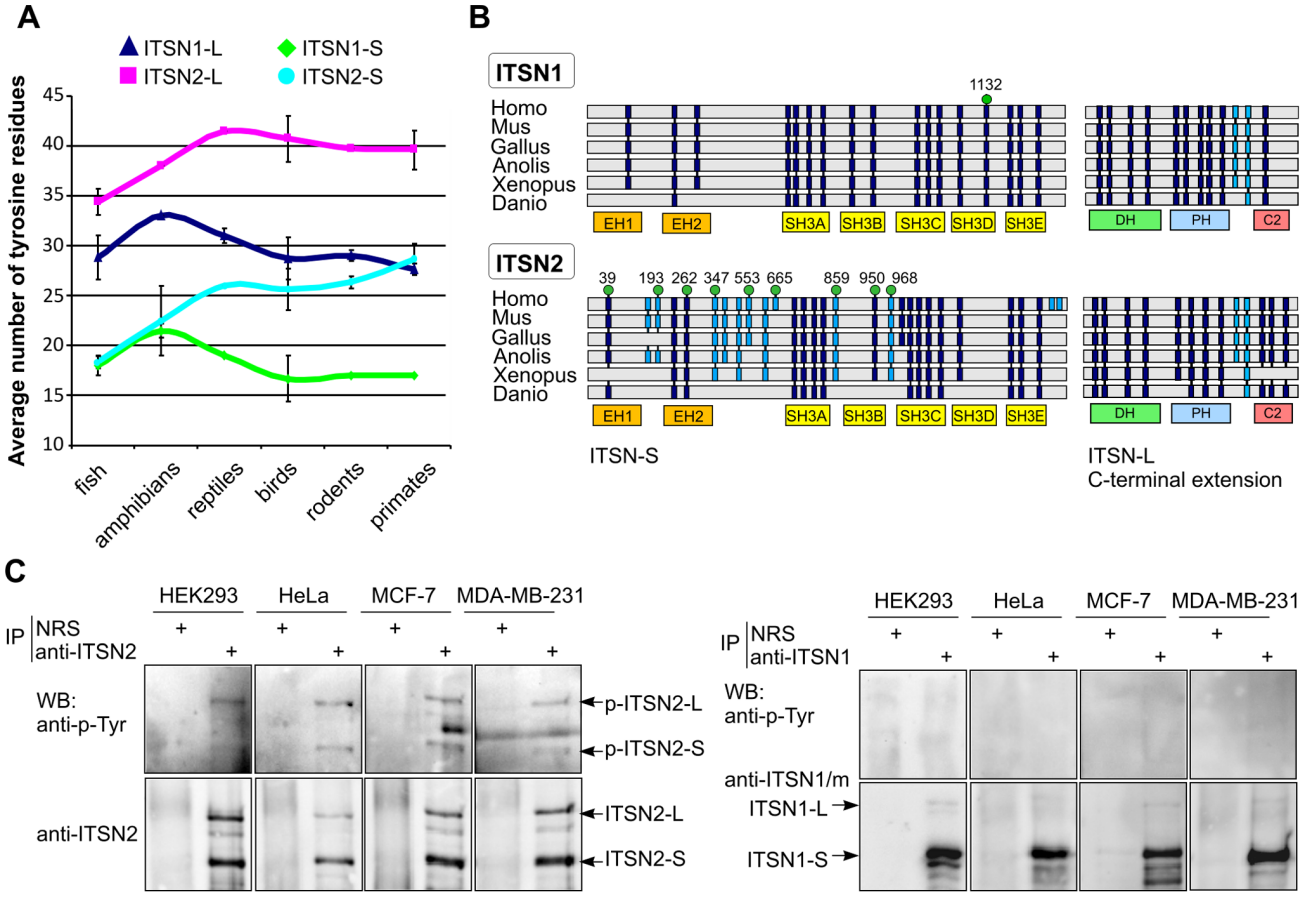
ITSN2 is phosphorylated on tyrosine residues. (A) Line graphs show the total number of tyrosine residues in ITSN1 and ITSN2 in a range of vertebrate taxonomic groups. Amino acid sequences of ITSN1 and ITSN2 orthologues in fish (*Danio rerio, Oreochromis niloticus* and *Tetraodon nigroviridis*), amphibians (*Xenopus tropicalis* and *Xenopus laevis*), reptiles (*Anolis carolinensis*), birds (*Gallus gallus, Meleagris gallopavo* and *Taeniopygia guttata*), rodents (*Mus musculus, Rattus norvegicus* and *Cricetulus griseus*) and primates (*Homo sapiens, Pan troglodita* and *Pongo abelii*) were obtained from the NCBI protein database (http: //ncbi.nlm.nih.gov). The average number of tyrosine residues per taxon is plotted, and error bars represent standard deviations. (B) Schematic representation of ITSNs domain organization and distribution of conserved tyrosine residues in ITSN1 and ITSN2 of various vertebrates. Tyrosine residues located within domain and interdomain regions are shown as dark blue and light blue boxes, respectively. Tyrosine residues that could be phosphorylated according to phosphoproteomic data (www.phosphosite.org) are indicated by green circles. The number above each circle indicates the position of the residue in the amino acid sequence of human ITSN. Abbreviations are defined as follows: Homo, *Homo sapiens*; Mus, *Mus musculus*; Gallus, *Gallus gallus*; Anolis, *Anolis carolinensis*; Xenopus, *Xenopus laevis*. (C) Lysates of growing HEK293, HeLa, MCF-7 and MBA-MB-231 cells were subjected to immunoprecipitation with rabbit polyclonal anti-ITSN2 (left panel) or anti-ITSN1 (right panel) antibodies. Normal rabbit serum (NRS) was used as control. Precipitated proteins were analyzed by Western blotting using anti-phosphotyrosine antibodies and antibodies against ITSN1 or ITSN2. The experiments were repeated at least twice with reproducible results. IP, immunoprecipitation; WB, Western blotting.

Given identified similarities between the ITSNs, we searched for novel binding interfaces that could provide specific protein-protein interactions. Sequence analysis revealed that ITSNs differ in the number of tyrosine residues and their location within the molecule. Comparative analysis of the ITSNs amino acid sequences of various vertebrates revealed that in primates the average number of tyrosines in ITSN1-S is seventeen compared to twenty-nine in ITSN2-S. Interestingly, in fish, the number of these residues in ITSN1-S and ITSN2-S is similar although during evolution the number of tyrosines has increased in ITSN2-S. A similar tendency was observed when number of tyrosine residues in ITSN1-L was compared to that of ITSN2-L (Figure 4A).

The distribution of conserved tyrosine residues in each ITSN was investigated (Figure 4B). Multiple sequence alignments demonstrated that all conserved residues in ITSN1-S were found in regions constituting its domains. In ITSN2-S, twelve conserved tyrosine residues located in interdomain regions, evolved later and most could be phosphorylated in a wide range of cell and cancer types according to phosphoproteomic data deposited in the PhosphositePlus database [44]. Within C-terminal regions specific for ITSN1-L and ITSN2-L no significant differences in the distribution of conserved tyrosine residues were observed.

To validate tyrosine phosphorylation of ITSN2, immunoprecipitation experiments were carried out with anti-ITSN2 antibodies using HEK293, HeLa, MCF-7 and MDA-MB-231 cell lysates. The samples were probed with anti-phosphotyrosine antibodies. The results demonstrated tyrosine phosphorylation of the ITSN2 short and long isoforms in all the cell lines tested (Figure 4C, left panel). In contrast no signal was detected with anti-phosphotyrosine antibodies in immunoprecipitates of ITSN1-S using the same lysates (Figure 4C, right panel).

Thus, during evolution ITSN2 acquired additional tyrosine residues in comparison to ITSN1 and undergoes tyrosine phosphorylation in the cell lines studied.

### Phosphorylation of ITSN2 is Affected by EGF and Chlorpromazine Treatment

**Figure 5:**
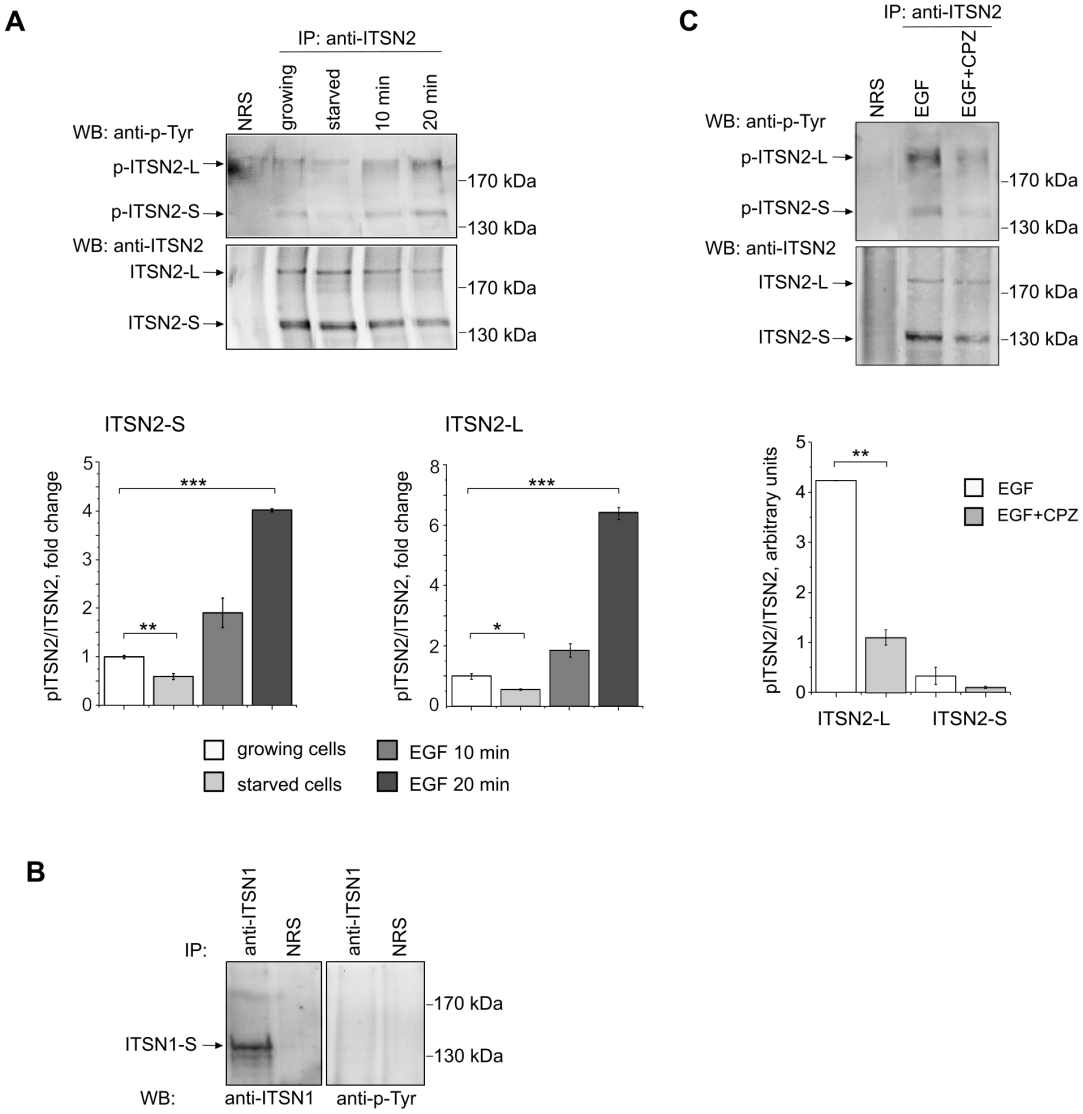
Tyrosine phosphorylation of ITSN2 depends on mitogenic stimulation and clathrin-mediated endocytosis. HeLa cells were maintained with 10% FBS, starved for 16 h or starved for 16 h and treated with 20 ng/ml of EGF for 10 and 20 min (A, B). For inhibition of endocytosis, serum-starved cells were treated with 30 µM chlorpromazine (CPZ) for 20 min followed by stimulation with EGF (20 ng/ml) in the presence of chlorpromazine (C). Extracts of cells were subjected to immunoprecipitation with antibodies against ITSN2 (A, C) and ITSN1 (B). Normal rabbit serum (NRS) was used as control. The precipitated proteins were analyzed by Western blotting with anti-phosphotyrosine, anti-ITSN2 and anti-ITSN1 antibodies. The ratio of pITSN2/ITSN2 intensity values was calculated, the data are presented as mean ±SEM (n = 3);*P<0.05,**P<0.01,***P<0.001. IP, immunoprecipitation; WB, Western blotting.

We investigated whether activation of mitogenic pathways could affect phosphorylation of ITSN2. For this, HeLa cells were serum-starved for 16 h and then treated with EGF. Immunoprecipitates of endogenous ITSN2 from growing, starved and EGF-stimulated cells were probed to investigate the level of phosphorylated proteins. The results obtained showed that the amount of phosphorylated ITSN2 isoforms increased after growth factor treatment and was reduced in serum-starved cells (Figure 5A). To study whether phosphorylation of ITSN1-S could be induced by EGF, immunoprecipitation was performed using the lysate of EGF-treated cells. In contrast to ITSN2 isoforms, no phosphorylation of ITSN1-S was detected (Figure 5B).

To determine whether phosphorylation of ITSN2 depends on internalization events, chlorpromazine was used to inhibit CME. In the micromolar range, this cationic amphipathic drug represses endocytosis of plasma membrane proteins [45]. Pretreatment of cells with chlorpromazine reduced EGF-induced phosphorylation of ITSN2-L (Figure 5C) suggesting that this modification depended on internalization. Difference in the levels of phosphorylation of ITSN2-S was not statistically significant.

### The SH2 Domains of Signaling Proteins Bind ITSN2

**Figure 6:**
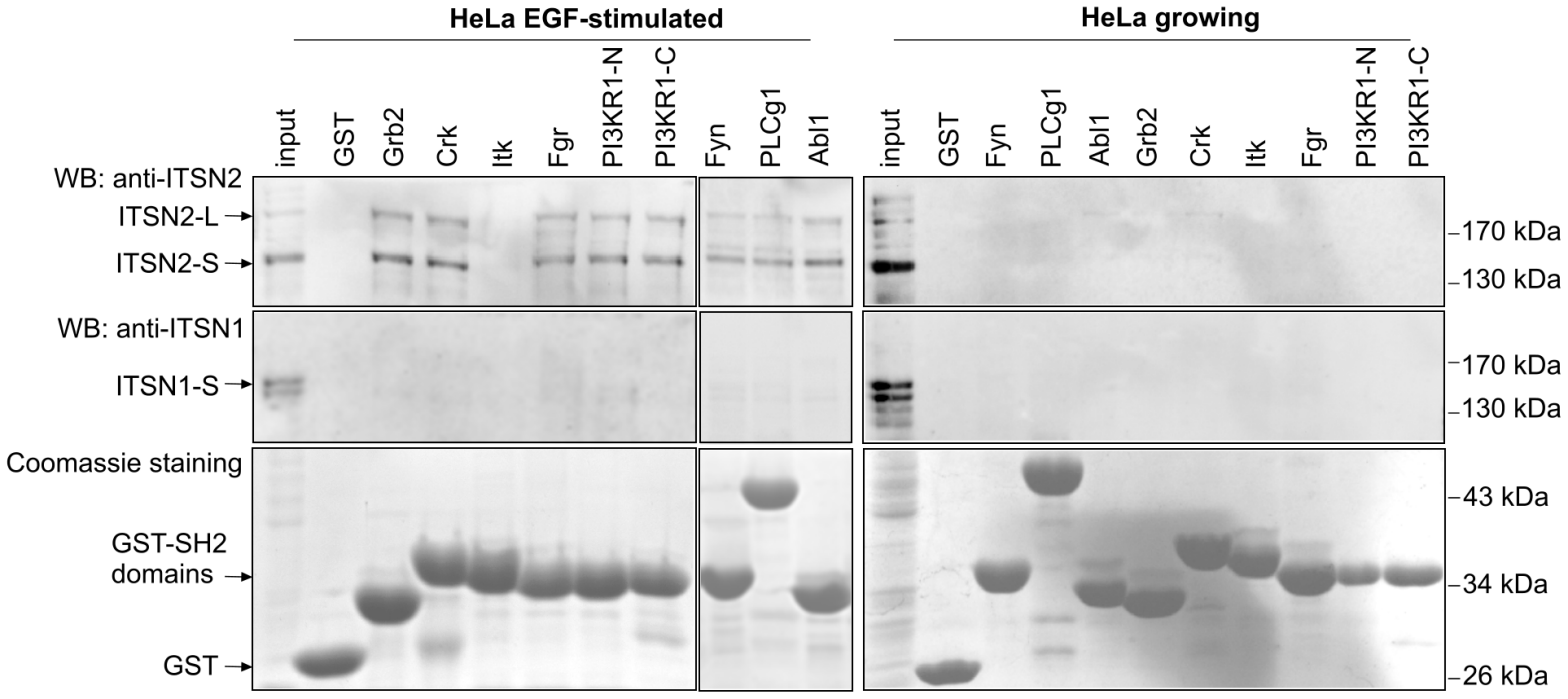
ITSN2 is specifically recognized by the SH2 domains of signaling proteins. HeLa cells were maintained in DMEM supplemented with 10% FBS, starved overnight and stimulated with 20 ng/ml EGF. Bacterially expressed and affinity purified GST-SH2 domains of Grb2, Crk, Itk, Fgr, Fyn, Abl1, PI3KR1, PLCg1 or GST alone (control) were bound to glutathione beads and used as bait to pull down ITSN proteins. For *in vitro* binding assays GST-fused proteins were incubated with lysates of EGF-treated (left panel) and growing (right panel) HeLa cells. Bound proteins were analyzed by Western blotting using antibodies against ITSN2. GST-fused proteins were visualized by Coomassie staining. The experiments were repeated three times with reproducible results. PI3KR1-N and PI3KR1-C correspond to the N- and C-terminal SH2 domains of PI3KR1, respectively. WB, Western blotting.

A search of specific “sensors” that could recognize the phosphorylated state of ITSN2 was performed. The SH2 domains of signaling proteins that potentially bind ITSN2 were determined using the Scansite service. Among them, we selected the SH2 domains that were predicted to bind tyrosine-based motifs phosphorylated according to phosphoproteomic data (www.phosphosite.org). A list of eight proteins involved in various cell pathways was obtained. The GST-tagged SH2 domains of Grb2, Crk, Itk, Fgr, Fyn, Abl1, PI3KR1 and PLCg1 were used for *in vitro* binding experiments. All the domains tested except Itk kinase effectively pulled down ITSN2 from the lysate of EGF-treated HeLa cells (Figure 6, left panel). It is worth mentioning that SH2/ITSN2 binding was not observed when growing HeLa cells were used (Figure 6, right panel). Recognition by the SH2 domains was specific only for ITSN2 and was not observed for ITSN1.

Taken together, the SH2 domains of several signaling proteins could specifically recognize phosphorylated ITSN2 isoforms.

## Discussion

The adaptor protein ITSN1 was extensively studied during almost one and a half decades. Initially, attention to this protein was paid due to the upregulation of its gene in patients with Down syndrome [9]. Recently, ITSN1 was suggested to contribute to Alzheimer’s disease and to the aggregation of huntingtin during Huntington’s disease [11], [13]. It is widely accepted that during early evolution of vertebrates, gene duplication caused the emergence of two highly similar genes that encode ITSN1 and ITSN2 [26]. So far these proteins were investigated independently except for a recent study of Henne et al. [2]. However, a comparison of ITSN1 and ITSN2 is of current importance to understand to what extent they could substitute for each other under normal and pathological conditions. Here we performed a comparative study of ITSNs and tried to follow their evolutionary diversification, and for the first time report evidence of post-translational regulation of ITSN2.

Previously, it was shown that ITSN1 and ITSN2 are coexpressed in most mammalian tissues [3], [4]. Intracellular colocalization of overexpressed recombinant ITSNs was also demonstrated [26], [35]. We now provide evidence concerning the subcellular codistribution of endogenous ITSNs and their association in cells (Figure 1B, C). These data indicate that ITSN1 and its paralogue ITSN2 are components of common protein complexes and have a predominantly common intracellular distribution in the cell line studied. Previously, ITSN1 was suggested to be constitutively associated with the endocytic adaptor Eps15 in a protein complex [3]. Presumably, ITSN1 and ITSN2 are predominantly engaged in this scaffolding complex that contains not only the ITSN1/Eps15 proteins as suggested initially. Recent data of Henne and colleagues demonstrated that four proteins, ITSN1/ITSN2 and Eps15/Eps15R, are required for FCHo1/2 clustering during clathrin-coated pit nucleation [2]. This complex can provide considerable recruitment platform where the regulator of the actin cytoskeleton ITSN2-L could be engaged in all cell types and ITSN1-L in neurons.

Consequently, we examined whether ITSN2 could bind protein partners of ITSN1. Most known ITSN1 interactors are ligands of its five SH3 domains (reviewed in [5]). A comparison of the SH3 domain structures of ITSNs showed a high similarity of their ligand-binding sites. Amino acid mismatches within respective SH3 domain pairs were predominantly located beyond the ligand-binding sites (Figure 2A). There are several possible explanations for this phenomenon. First, selection kept the ligand binding sites of the ITSNs SH3 domains similar whereas amino acid residues beyond the sites varied to a greater extent. Another explanation concerns noncanonical functions of the SH3 domains and the existence of additional interaction interfaces. Recently, the SH3E domain of ITSN1 was shown to bind its DH domain via the surface opposite the PXXP-binding groove and provide inhibition of GTP-exchange activity [46]. There are data demonstrating the ability of homodimerization and heterodimerization of the SH3 domains. The SH3 domains of the adaptor protein CRKL homodimerize to regulate exposure of the nuclear export signal [47]. Heterodimerization of the Vav and Grb2 SH3 domains was shown to be important for the activation of Vav exchange activity [48]. Thus, diversification of major ITSNs protein-binding regions could occur not in proline-binding sites but rather within surfaces that are involved in recognition of unknown targets.

The results from *in vitro* binding experiments demonstrate the ability of the SH3 domains of ITSN2 to pull down all the protein partners of ITSN1 investigated here. Moreover, novel SH3 ligands, the endocytic adaptor POB1 and the signaling protein Sema6A, were common to both ITSNs (Figure 3).

We intended to search for a putative novel protein-interaction interface that could be distinct between ITSN molecules. A comparison of the primary sequences of the ITSNs demonstrated that ITSN2-S contains 60% more tyrosine residues than ITSN1-S. Accumulation of tyrosines in ITSN2 isoforms was clearly observed in the range of its vertebrate orthologues from fish to primates. All the conserved tyrosine residues of ITSN1-S were located within protein domains whereas additional tyrosine residues in ITSN2-S were found in interdomain regions. Tyrosine phosphorylation of ITSN2 isoforms but not ITSN1-S was detected in various cell lines. This is in good correspondence with phosphoproteomic data about this modification of ITSNs. PhosphositePlus database contains 1601 references reporting tyrosine phosphorylation of ITSN2 and only 3 for ITSN1. Phosphorylation of amino acid residues Y553 and Y968 of ITSN2 was demonstrated in 772 and 700 references, respectively, strongly arguing for their modification. It is notable that phosphorylation of Y968 depends on EGFR activation [49]. These data are in line with our observation of enhancement of ITSN2 phosphorylation in response to EGF treatment. Thus, based on our results together with high-throughput mass spectrometry data it is possible to assume that ITSN2 isoforms undergo more intense tyrosine phosphorylation than could ITSN1-S. Recently it was demonstrated that under specific conditions, such as coexpression of viral LMP2A (latent membrane protein 2A) protein together with Syk kinase, ITSN1-S could be tyrosine phosphorylated in HEK293 cells [37]. The pattern of phosphorylation could be different in various tissues. ITSN1-L has a role in specialized neuronal cell functions and is expressed at high levels in these cells [21]–[23]. The cell lines used in this study demonstrate barely detectable expression of this ITSN1 isoform. Therefore, the possibility of ITSN1-L phosphorylation should be investigated in neuronal cells. In favour of this, there exist phosphoproteomic data presenting no phosphopeptides of ITSN1-L in cell lines studied.

Acquisition by ITSN2 of additional tyrosine residues and their posttranslational modification lead us to assume that tyrosine-based linear motifs emerged during evolution to regulate ITSN2. Evolution of linear motifs is considered to be the fastest and major mechanism involved in changing protein interaction networks [50]. One could expect that phosphorylation of the linear motif allows recognition of the motif by proteins bearing phosphotyrosine-binding domains. We have demonstrated that the SH2 domains of the kinases Fyn, Fgr and Abl1, the regulatory subunit of PI3K, the adaptor proteins Grb2 and Crk, and phospholipase C gamma could mediate binding to ITSN2. In spite of phosphorylation of ITSN2 isoforms in growing cells, interactions with the SH2 domains were detected only in EGF-stimulated cells. It could be suggested that EGF treatment induces specific phosphorylation of motifs recognized by certain SH2 domains.

Taken together, specific tyrosine phosphorylation of ITSN2 and its recognition by SH2-containing proteins could be one of the key elements to understand ITSNs functional segregation in cellular pathways. Identification of the functional consequences of the interactions observed is a challenge for further investigations.

## Supporting Information

Figure S1 **Characterization of antibodies against ITSN1 and ITSN2.** (A) Lysates of HEK293 cells were resolved by SDS-PAGE with subsequent immunoblot analysis using rabbit anti-ITSN1, mouse anti-ITSN1/m or rabbit anti-ITSN2 antibodies. Antigen competition assay for anti-ITSN2 (B) and anti-ITSN1 (C) antibodies was performed. Lysate of HEK293 cells was subjected to immunoblotting using the indicated antibodies (first lane) or antibodies preincubated with the respective immunogen (second lane). The immunogen was used with 50-fold molar excess.
(TIF)

Figure S2 **Overexpressed ITSNs are colocalized and associated in a protein complex in HEK293 cells.** (A) HEK293 cells were cotransfected with Omni-ITSN2-S and mCherry-ITSN1-S. ITSN2-S was stained with anti-Omni antibodies and visualized with Alexa 488-conjugated secondary antibodies. Scale bar: 10 µm. (B) Lysates of HEK293 cells expressing Omni-ITSN2-S were subjected to immnunoprecipitation using anti-Omni antibodies. Immunoprecipitates were probed with antibodies against ITSN1 or tag. Normal rabbit serum (NRS) was used as control.
(TIF)

Figure S3 **Amino acid mismatches within ligand-binding sites of the SH3 domains of ITSNs are conserved.** Multiple alignments of protein sequences of the ITSNs SH3 domains. Abbreviations are defined as follows: Homo, *Homo sapiens*; Bos, *Bos taurus*; Mus, *Mus musculus*; Gall, *Gallus gallus*; Anol, *Anolis carolinensis*; Xen, *Xenopus laevis*. The SH3 domains of ITSN1 and ITSN2 are indicated as A1–E1 and A2–E2, respectively. The alignment was generated using the ClustalW algorithm. Identical residues are highlighted in red, homologous amino acids are shown in red letters. Amino acid residues that form ligand-binding sites of the SH3 domains are indicated by boxes, and mismatches within these regions are shown by boxes of different colours.
(TIF)
