# Supplementary figures and images for: Adaptor Proteins Intersectin 1 and 2 Bind Similar Proline-Rich Ligands but Are Differentially Recognized by SH2 Domain-Containing Proteins
Source: PLoS One. 2013 Jul 25;8(7):e70546. doi: 10.1371/journal.pone.0070546 (PMC3723668; doi:10.1371/journal.pone.0070546)

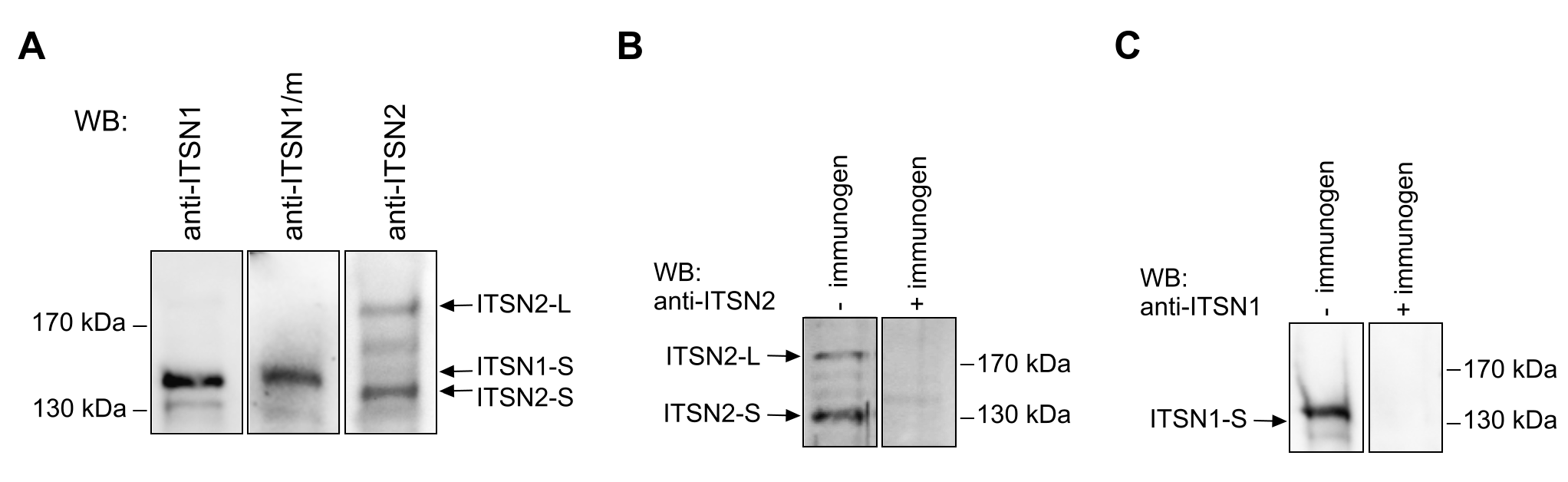

Supplement: Figure S1 — Characterization of antibodies against ITSN1 and ITSN2. (A) Lysates of HEK293 cells were resolved by SDS-PAGE with subsequent immunoblot analysis using rabbit anti-ITSN1, mouse anti-ITSN1/m or rabbit anti-ITSN2 antibodies. Antigen competition assay for anti-ITSN2 (B) and anti-ITSN1 (C) antibodies was performed. Lysate of HEK293 cells was subjected to immunoblotting using the indicated antibodies (first lane) or antibodies preincubated with the respective immunogen (second lane). The immunogen was used with 50-fold molar excess. (TIF) [file pone.0070546.s001.tif]

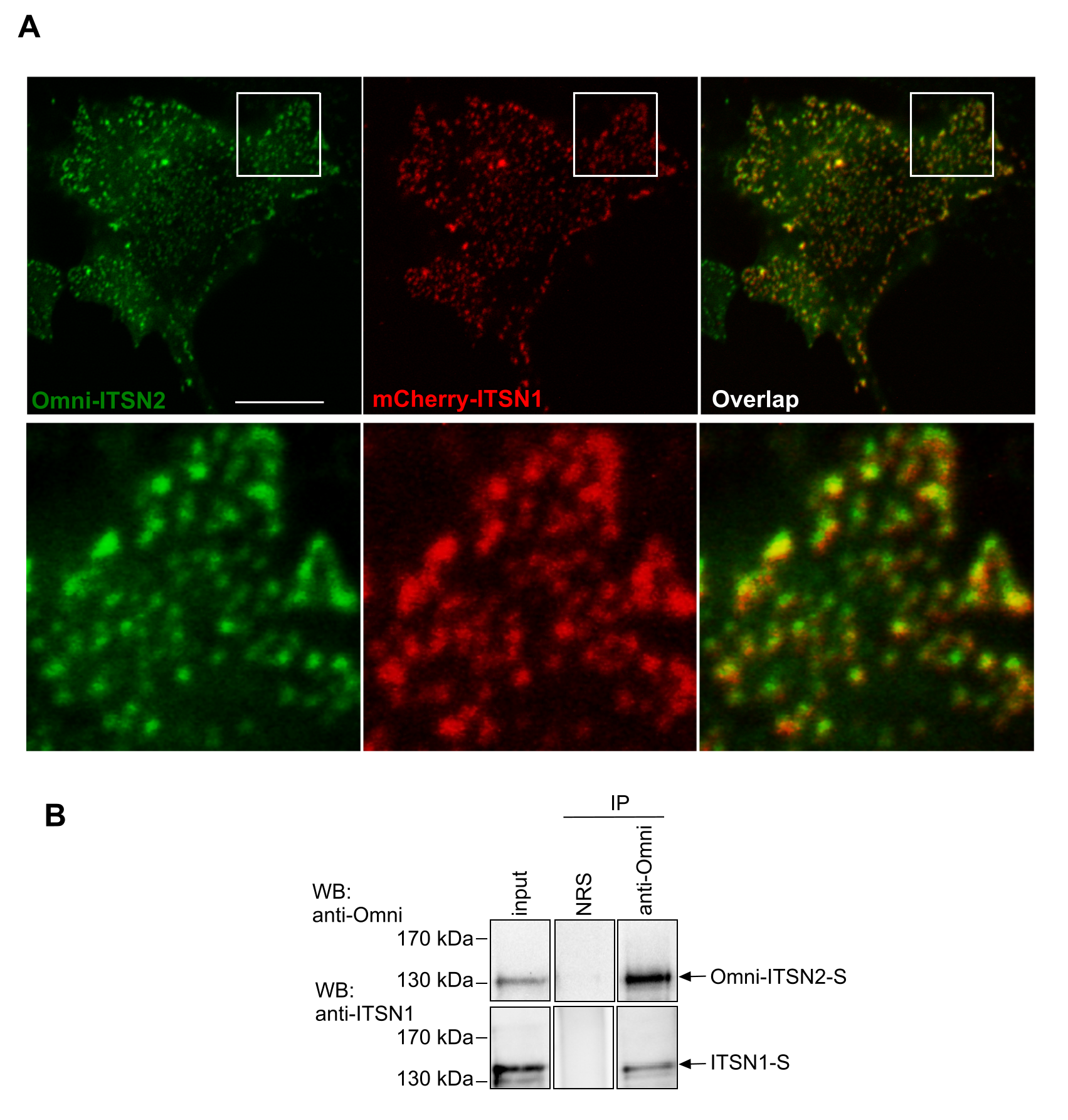

Supplement: Figure S2 — Overexpressed ITSNs are colocalized and associated in a protein complex in HEK293 cells. (A) HEK293 cells were cotransfected with Omni-ITSN2-S and mCherry-ITSN1-S. ITSN2-S was stained with anti-Omni antibodies and visualized with Alexa 488-conjugated secondary antibodies. Scale bar: 10 µm. (B) Lysates of HEK293 cells expressing Omni-ITSN2-S were subjected to immnunoprecipitation using anti-Omni antibodies. Immunoprecipitates were probed with antibodies against ITSN1 or tag. Normal rabbit serum (NRS) was used as control. (TIF) [file pone.0070546.s002.tif]

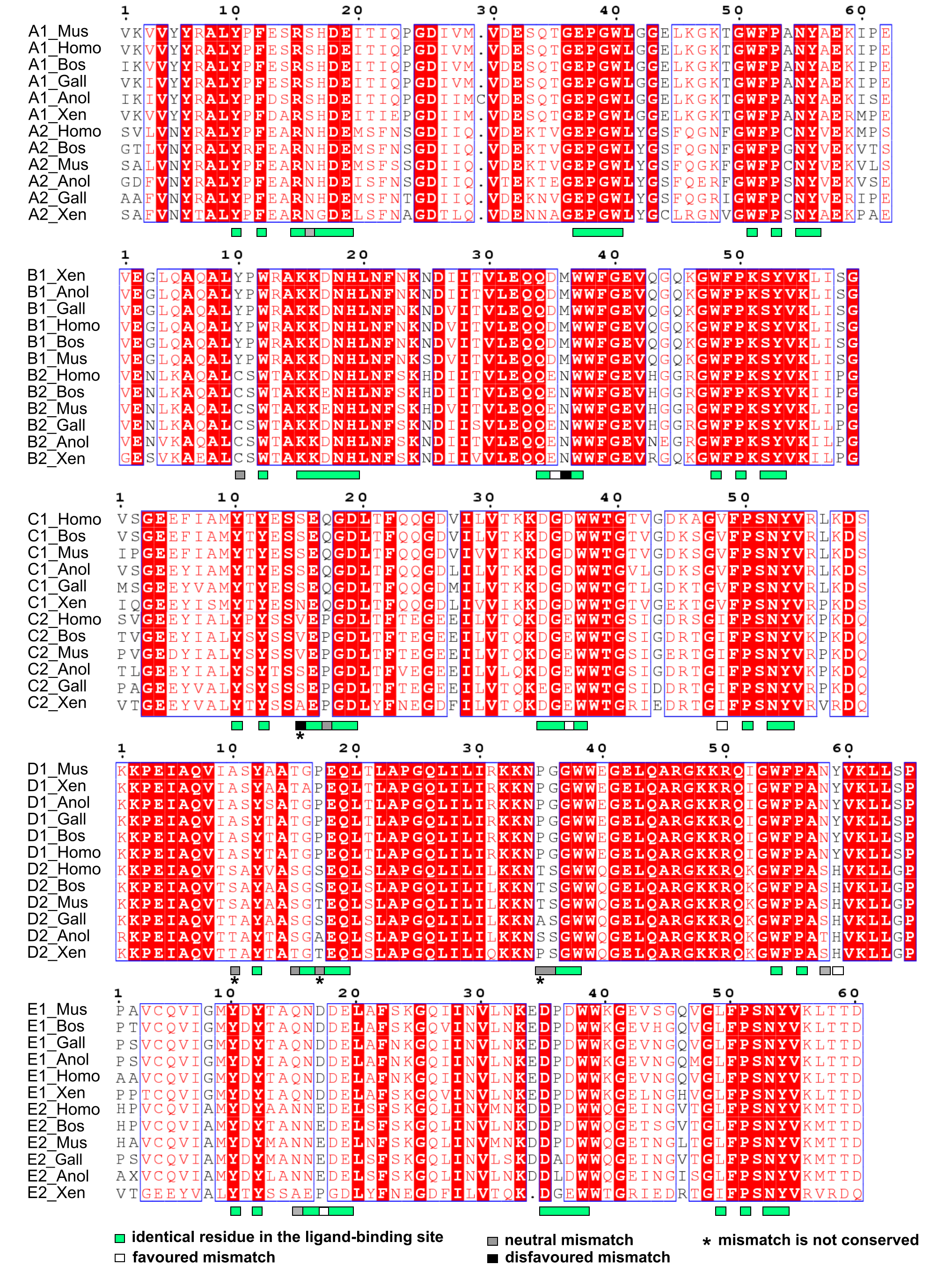

Supplement: Figure S3 — Amino acid mismatches within ligand-binding sites of the SH3 domains of ITSNs are conserved. Multiple alignments of protein sequences of the ITSNs SH3 domains. Abbreviations are defined as follows: Homo, Homo sapiens; Bos, Bos taurus; Mus, Mus musculus; Gall, Gallus gallus; Anol, Anolis carolinensis; Xen, Xenopus laevis. The SH3 domains of ITSN1 and ITSN2 are indicated as A1–E1 and A2–E2, respectively. The alignment was generated using the ClustalW algorithm. Identical residues are highlighted in red, homologous amino acids are shown in red letters. Amino acid residues that form ligand-binding sites of the SH3 domains are indicated by boxes, and mismatches within these regions are shown by boxes of different colours. (TIF) [file pone.0070546.s003.tif]
